# Supplementary material for: Decolourization of crystal violet using nano-sized novel fluorite structure Ga2Zr2−xWxO7 photocatalyst under visible light irradiation
Source: R Soc Open Sci. 2020 Mar 11;7(3):191632. doi: 10.1098/rsos.191632 (PMC7137949; doi:10.1098/rsos.191632)
Supplement: supplementary information [file rsos191632supp1.docx]

Calcination

T=600 °C, 2h

T=1000 ºC, 24h

Calcination

T=500 °C, 2h

T=600 ºC, 24h

Grinding

Charring

T=300 °C

Grinding

Gel

Mix

T=140 °C

Ethylene glycol

H_2_O+NO_x_

Mix

CA/nitrates

1) ZrO(NO_3_)_3_.2H_2_O

3) Citric acid (CA)

2) Ga(NO_3_)_3_

Ga_2_Zr_2_O_7_powder

S. 1: Flow chart for the full process of polymeric precursor synthesis and preparation of Ga_2_Zr_2_O_7_ powder.


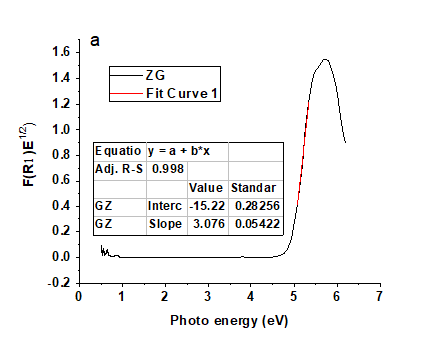

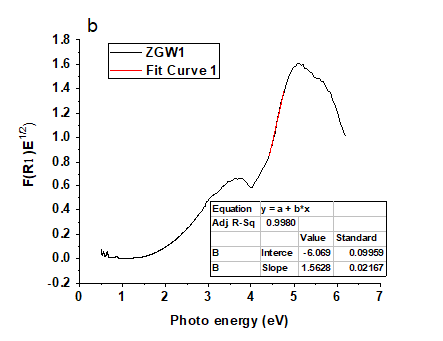


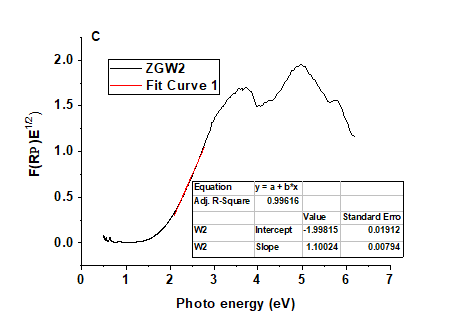

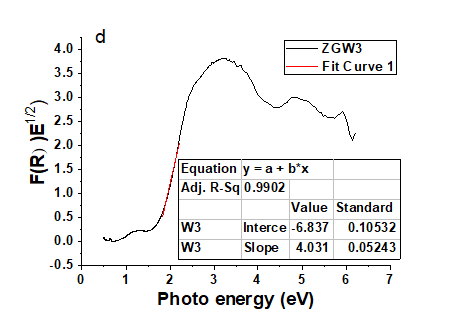

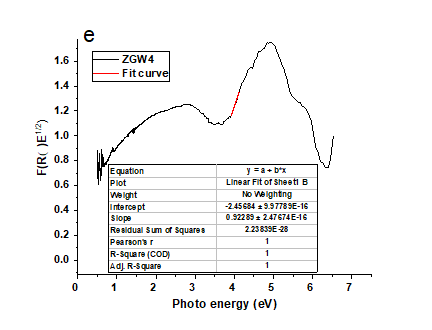


S. 2: The linear fit for the estimation estimated band gap values of ZG, ZGW1, ZGW2, ZGW3 and ZGW4 samples


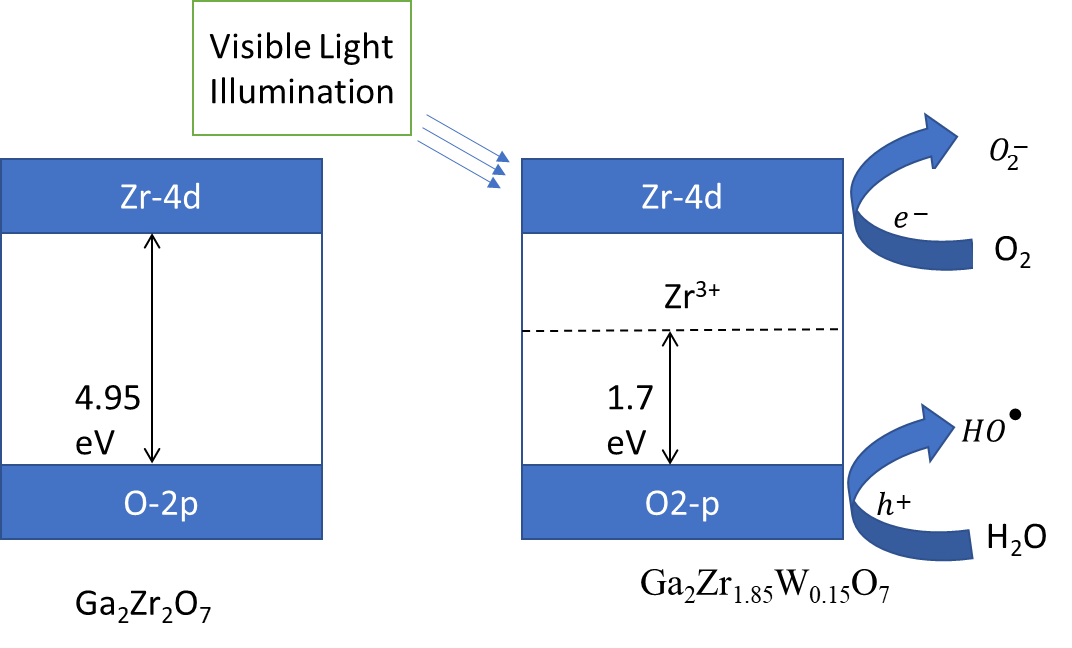


S. 3: schematic diagram for band gap and the proposed new energy levels introduced by doping for Ga_2_Zr_2-x_W_x_O_7_ systems. The arrow represents the band gap.


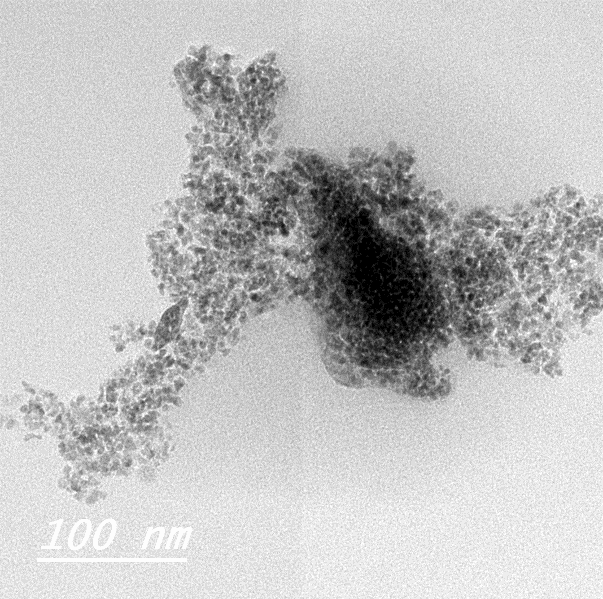

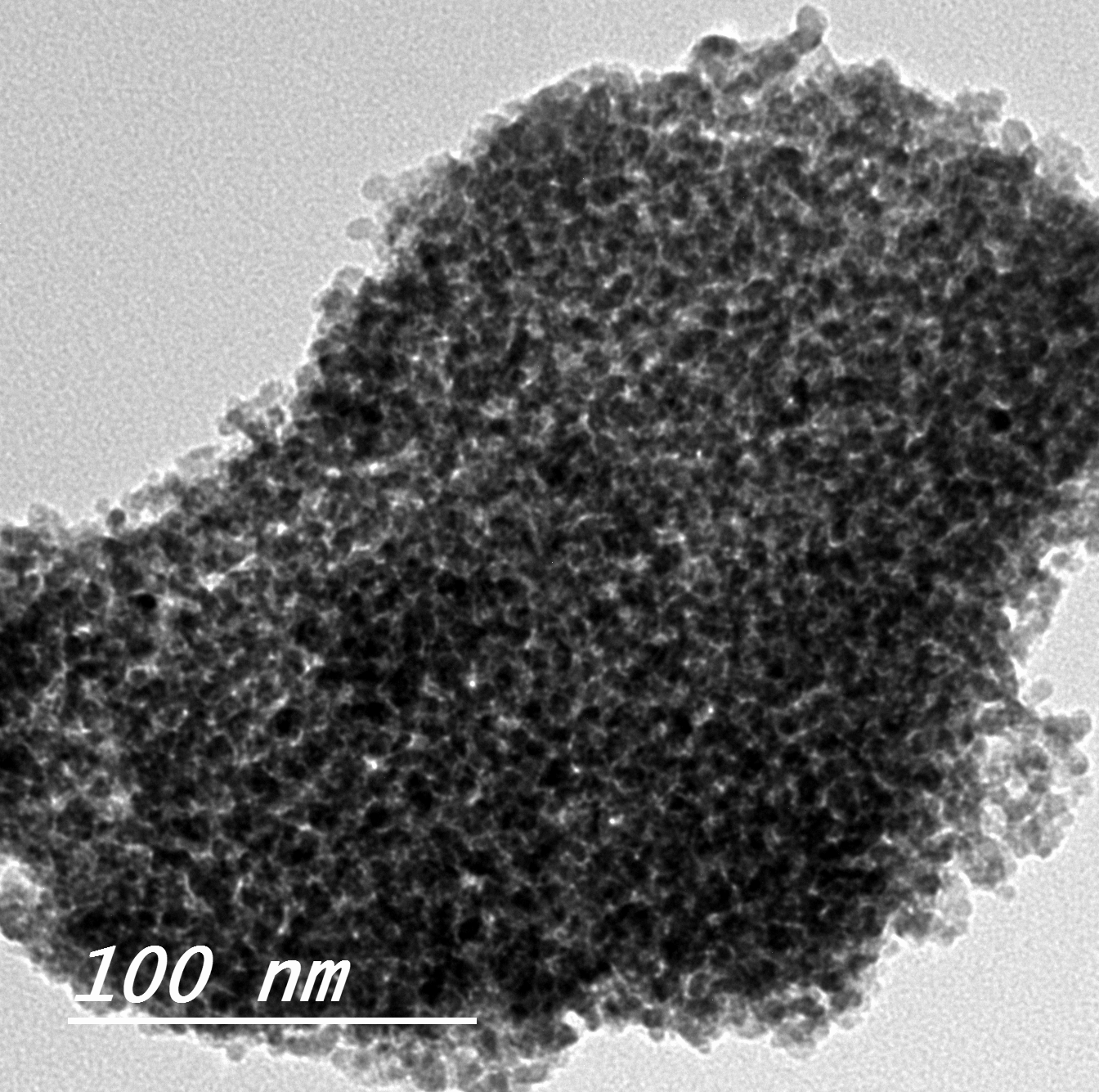


**aa**

**b**


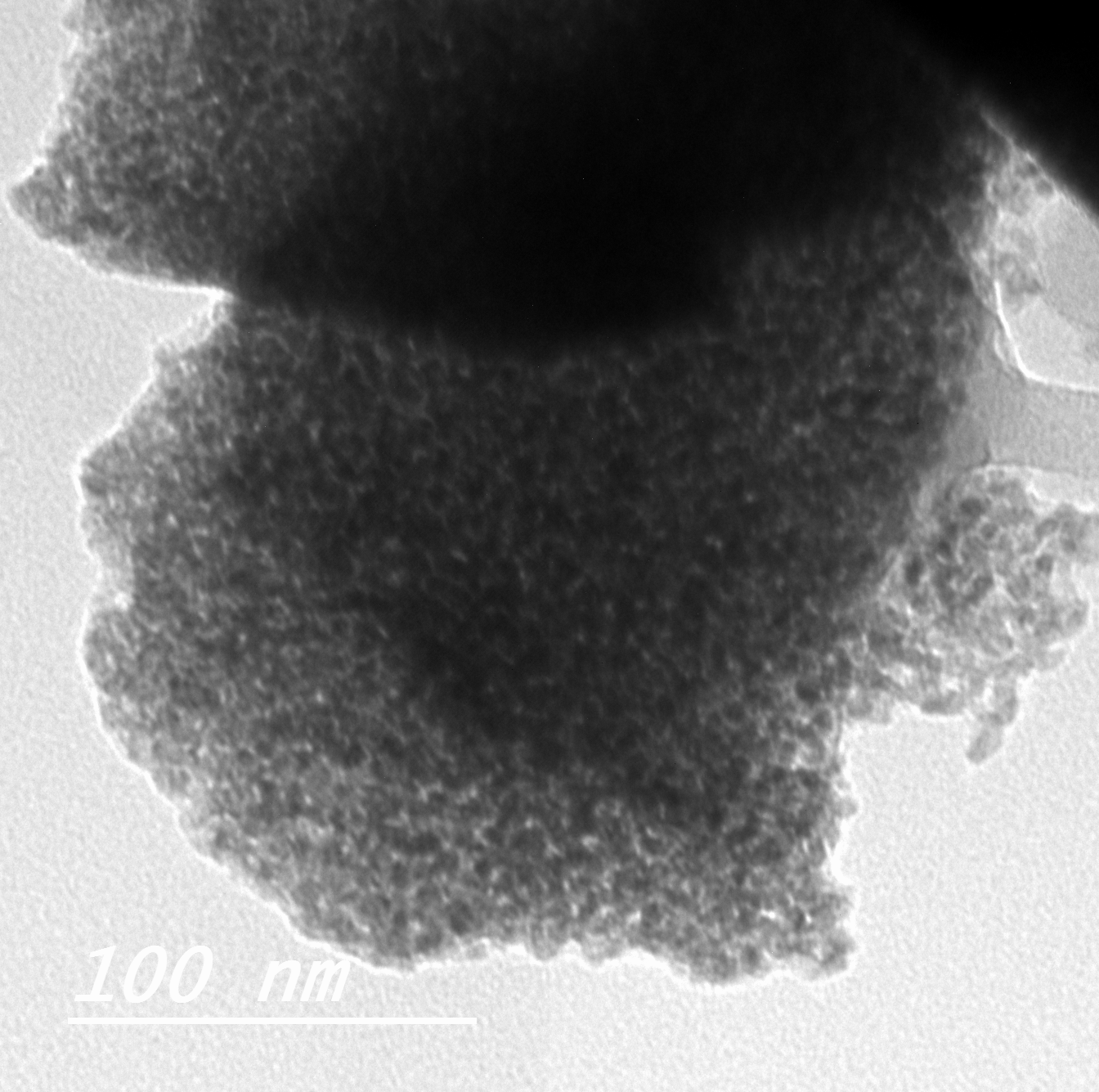

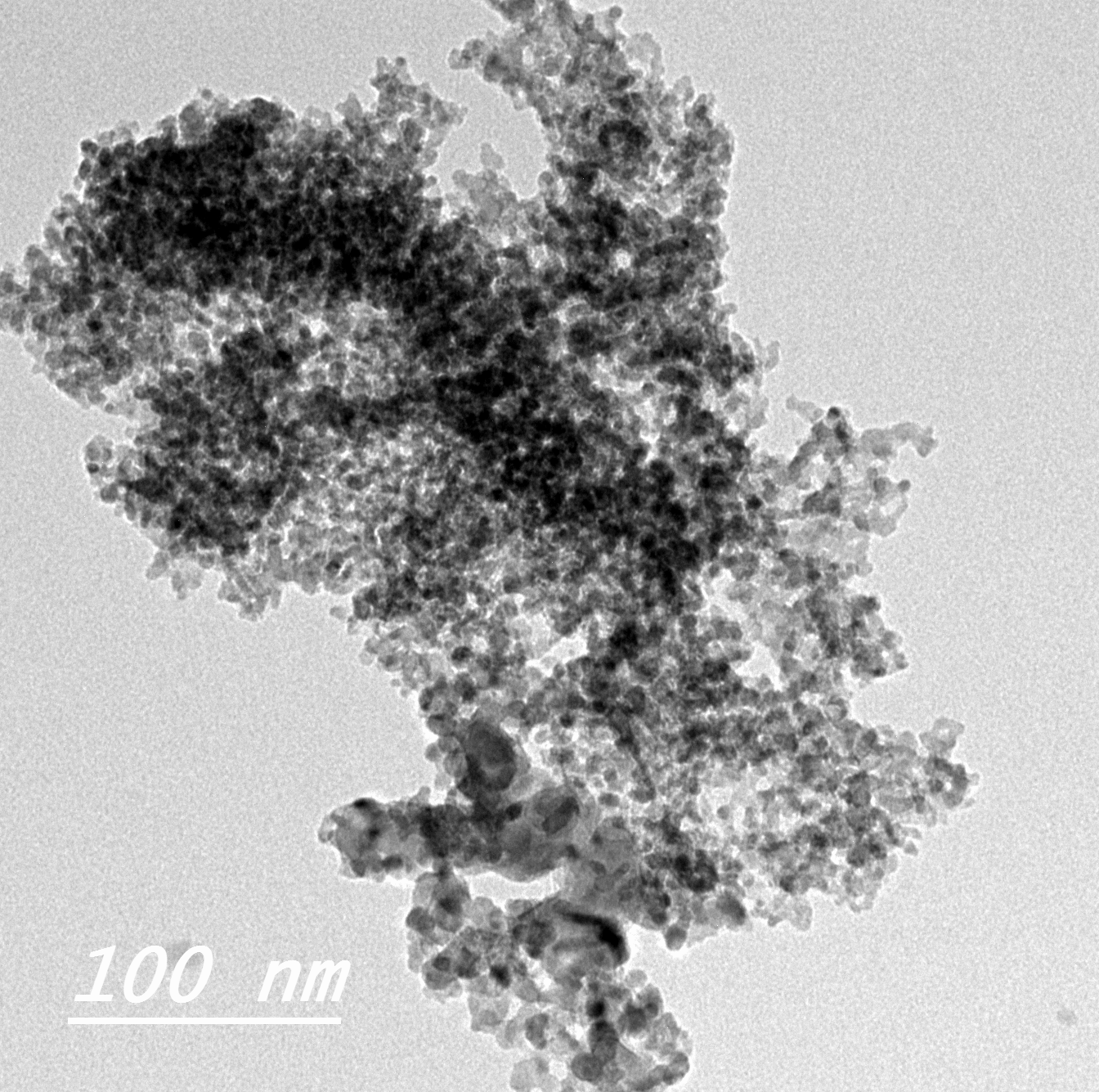


**c**

**d**

S. 4: TEM micrograph of a) ZG, b) ZGW1 and C) ZGW2 and d) ZGW3 samples calcined at 600°C/2h.


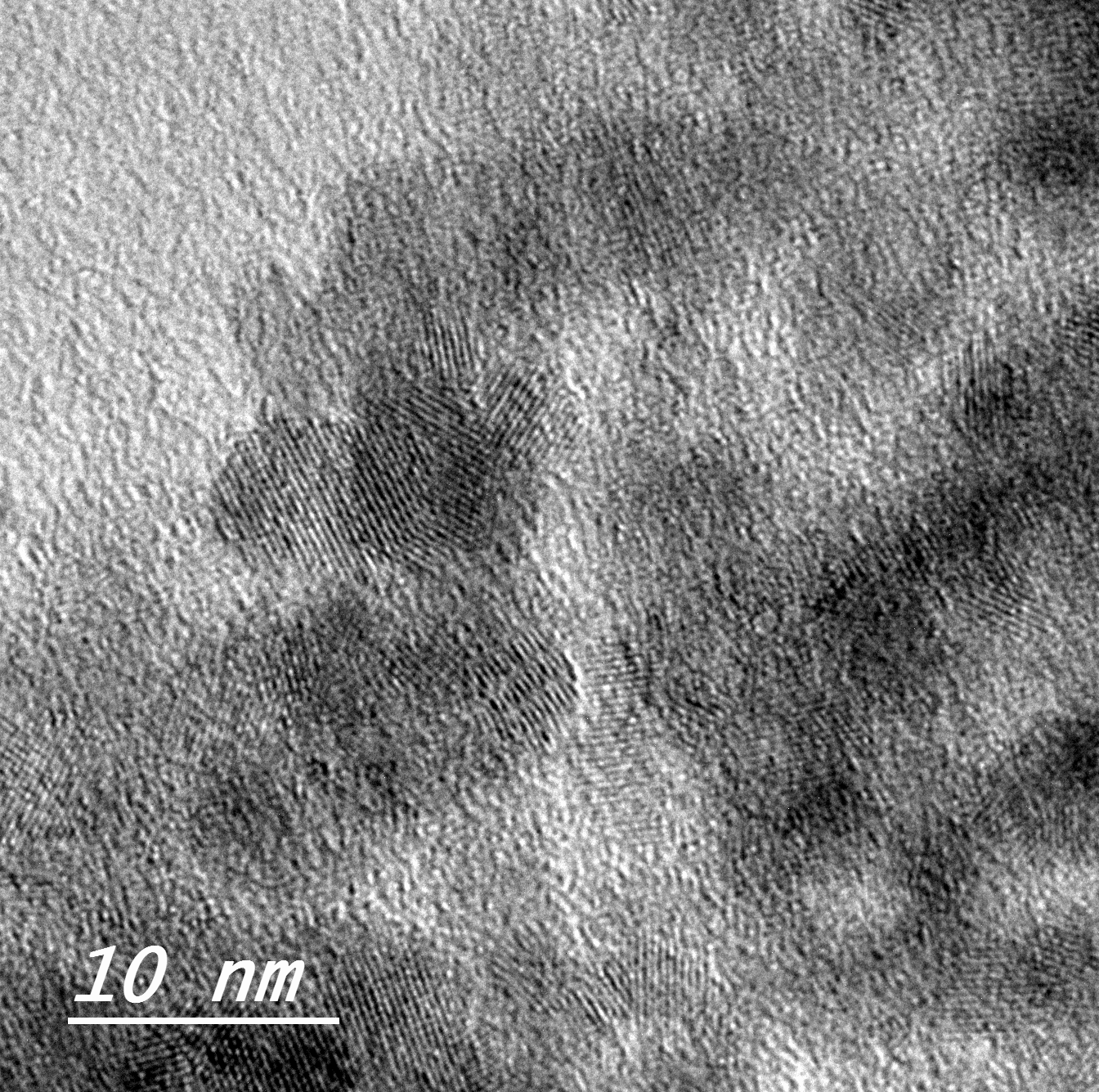

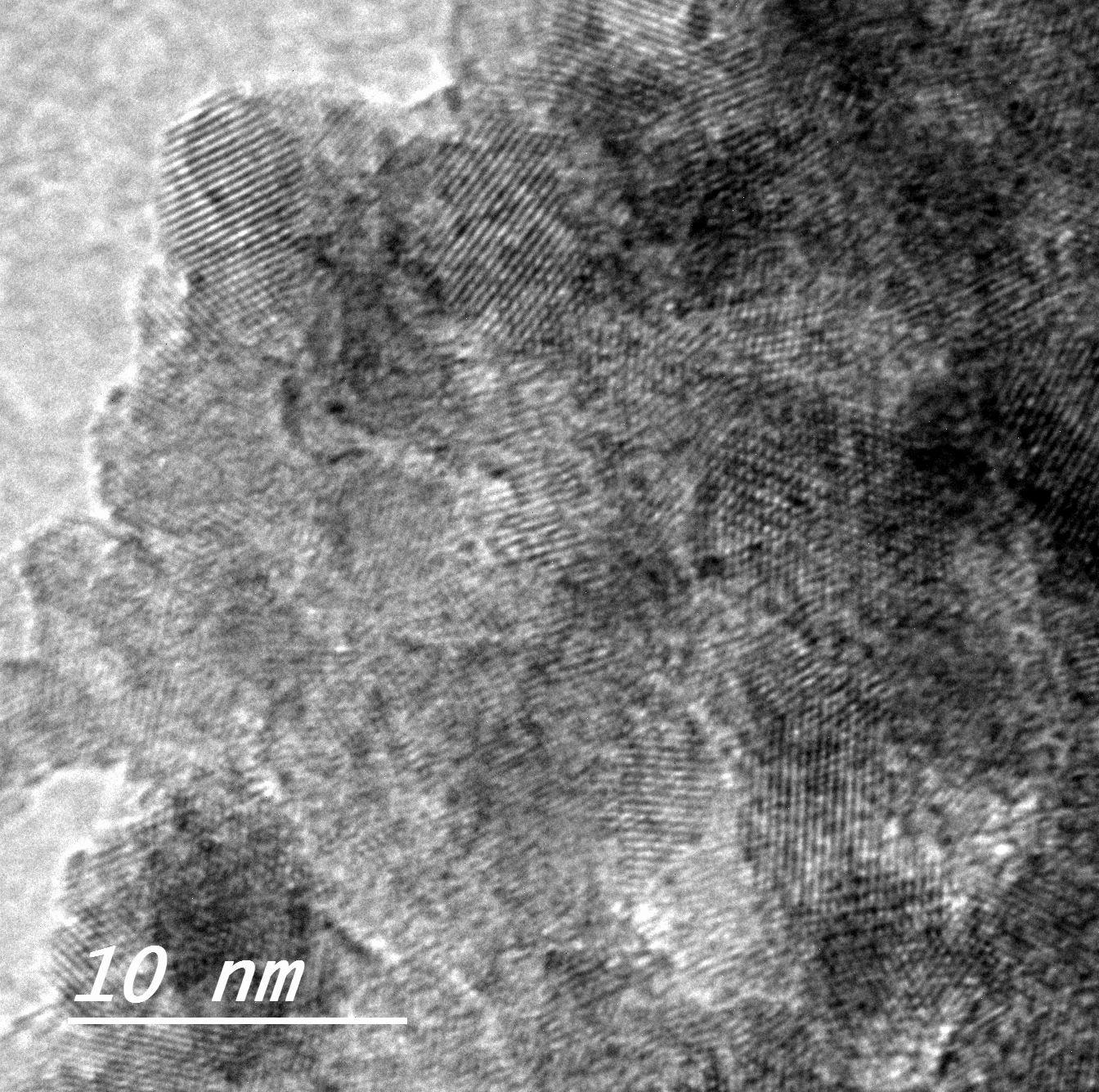

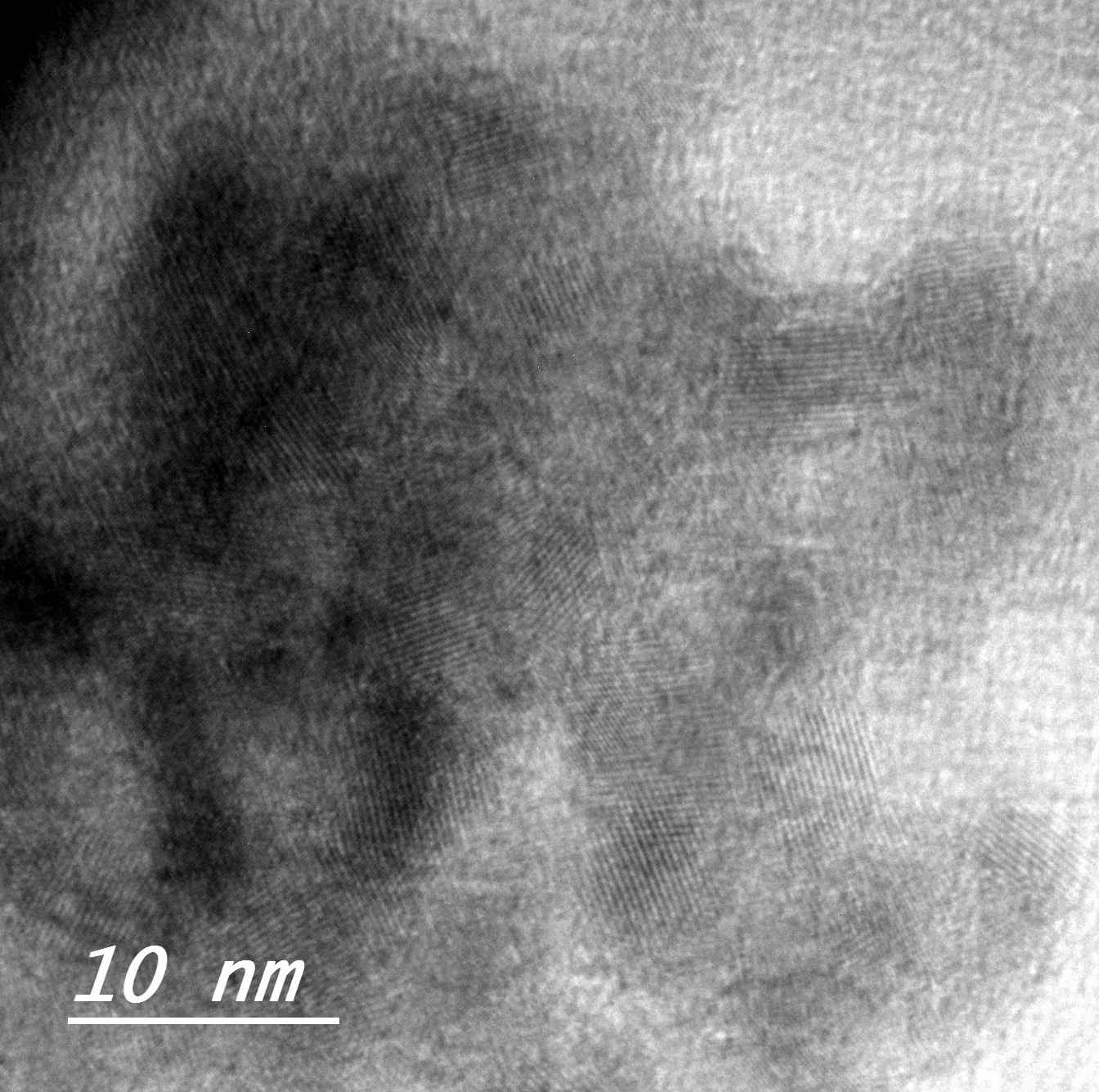

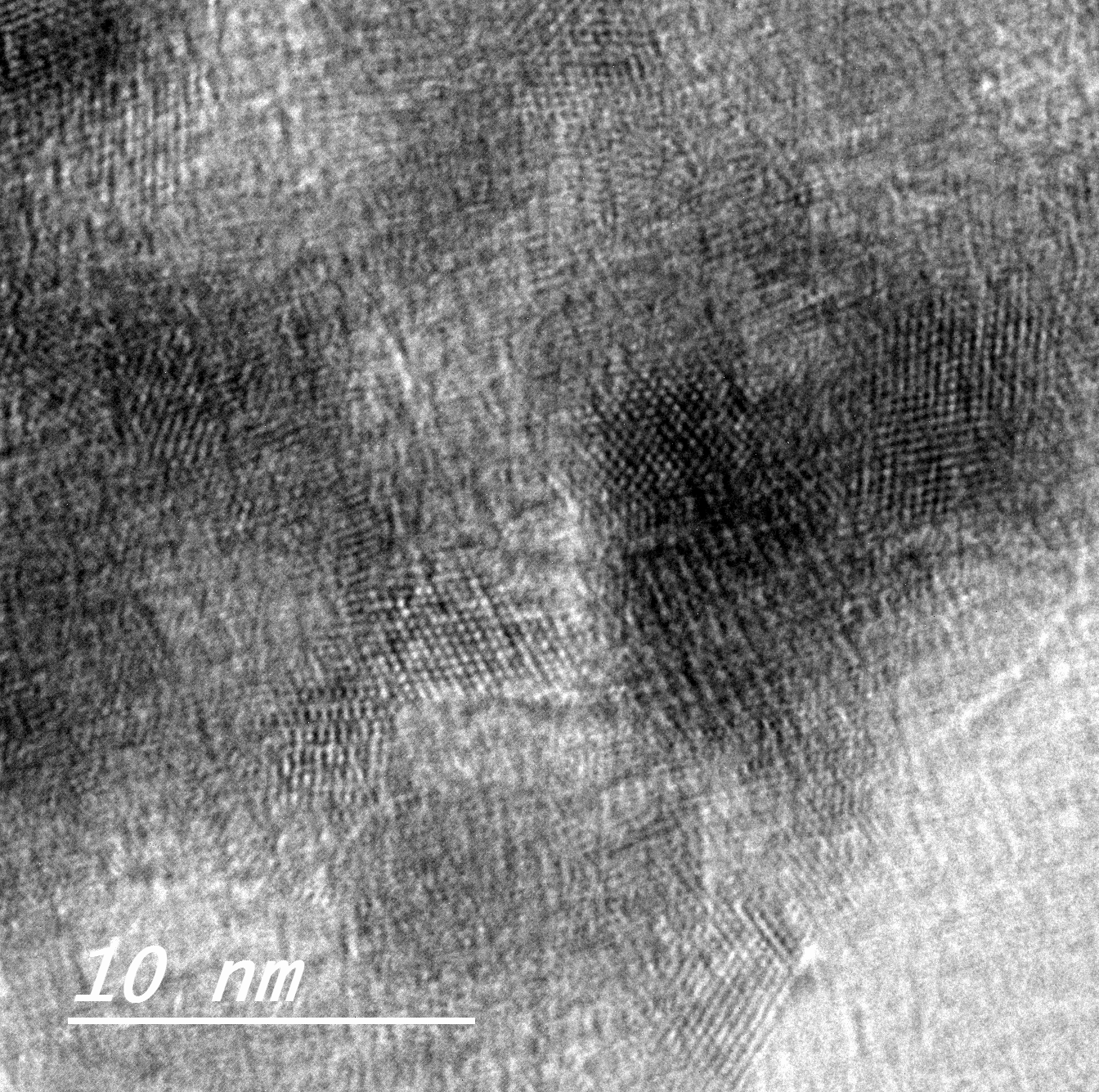


**d**

**c**

**b**

**a**

S. 5: HRTEM micrograph of a) ZG, b) ZGW1 and C) ZGW2 and d) ZGW3 samples calcined at 600 °C/2h.


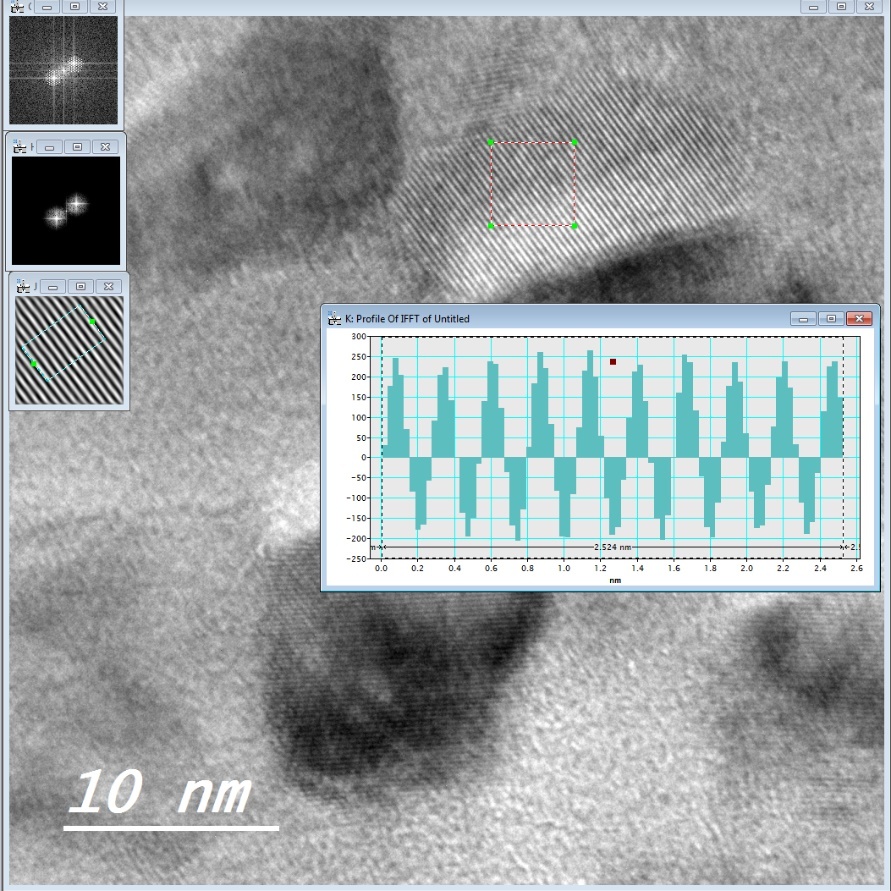


S. 6: HRTEM image of ZGW3 sample.
